# Utrophin Compensates dystrophin Loss during Mouse Spermatogenesis

**DOI:** 10.1038/s41598-017-05993-8

**Published:** 2017-08-07

**Authors:** Hung-Chih Chen, Yu-Feng Chin, David J. Lundy, Chung-Tiang Liang, Ya-Hui Chi, Paolin Kuo, Patrick C. H. Hsieh

**Affiliations:** 1 Institute of Biomedical Sciences, Academia Sinica, Taipei, 115 Taiwan; 2 National Laboratory Animal Center, National Applied Research Laboratories, Taipei, 115 Taiwan; 3 Institute of Biotechnology and Pharmaceutical Research, National Health Research Institutes, Zhunan, Miaoli County 350 Taiwan; 4 Department of Obstetrics & Gynecology, College of Medicine, National Cheng Kung University, Tainan, 701 Taiwan; 5 Institute of Medical Genomics and Proteomics, Institute of Clinical Medicine and Cardiovascular Surgery Division, National Taiwan University and Hospital, Taipei, 100 Taiwan

## Abstract

Duchenne muscular dystrophy (DMD) is an X-linked genetic disorder resulting from mutations in the *dystrophin* gene. The *mdx/utrn* ^−/−^ mouse, lacking in both dystrophin and its autosomal homologue utrophin, is commonly used to model the clinical symptoms of DMD. Interestingly, these mice are infertile but the mechanisms underlying this phenomenon remain unclear. Using *dystrophin* deficient *mdx* mouse and *utrophin* haplodeficient *mdx/utrn* ^+/−^ mouse models, we demonstrate the contribution of Dp427 (full-length dystrophin) and utrophin to testis and epididymis development, as well as spermatogenesis. We show that Dp427 deficiency disturbed the balance between proliferation and apoptosis of germ cells during spermatogenesis, which was further disrupted with *utrophin* haplodeficiency, deciphering a compensatory role of utrophin for dystrophin in the male reproductive system. In the spermatozoa, we have found a compensatory response of utrophin to dystrophin deficiency - namely the upregulation and relocation of utrophin to the flagellar midpiece. This study demonstrates the contribution of Dp427 and utrophin in male fertility, suggesting a potential pathology in DMD patients.

## Introduction

Lack of the protein dystrophin results in Duchenne muscular dystrophy (DMD), a devastating hereditary childhood disease. DMD is characterized by progressive muscle degeneration, loss of ambulation in adolescence, and cardiopulmonary failure that leads to the death of DMD patients during the third decades of their lives^1^. Dystrophin is encoded by the largest known human gene *DMD*, which spans about 2.4 Mb of Xp21 and comprises 79 exons^2, 3^. Dystrophin is a rod-shaped cytoskeletal protein linking extracellular laminin and intracellular F-actin in muscles to mediate force transmission and signal transduction^4–6^. In addition to this mechanical role, dystrophin also regulates asymmetric division of satellite cells by regulating the polarity of a microtubule kinase MARK2^7^. Alternative promoters and splicing produce tissue-specific isoforms of dystrophin, including the full length Dp427 in skeletal and cardiac muscles, Dp260 in retina, Dp140 in brain and kidney, Dp116 in peripheral nerves, and ubiquitous Dp71^8^. Although predominant in skeletal and cardiac muscles, Dp427 mRNA is also detectable in the mouse testes^9^.

Utrophin (product of *UTRN*), an autosomal homologue of dystrophin, maps to human chromosome 6 (6q24) and mouse chromosome 10^10^. Like dystrophin, utrophin contains an N-terminal actin-binding domain and a C-terminal dystroglycan-binding domain^11^. The nucleic acids and amino acids of utrophin respectively share 65% and 80% identity with dystrophin in human^10^. Utrophin is expressed ubiquitously in many fetal and adult tissues, including liver, spleen, skeletal muscles and testes^12^. Utrophin localizes in the neuromuscular junction of adult skeletal muscles, whereas it localizes to the sarcolemma in regenerating muscle fibers^13, 14^. Sarcolemma recruitment and upregulation of utrophin in the skeletal muscle of *mdx* mice, the most common animal model of DMD, with a nonsense mutation in exon 23, implies that utrophin plays a role to compensate for the loss of dystrophin^14–16^. A similar compensatory effect of utrophin has been proposed in DMD patients, who also have a higher level of utrophin in their skeletal muscles^17^.

Although dystrophin and utrophin are both expressed in the testes, their function in the male reproductive system remains unclear. Up71 (a short isoform of utrophin) and Dp71 are expressed in the postacrosomal region of the spermatozoa, with the expression of Dp71 extending to the midpiece of the spermatozoal flagella. The absence of Dp71 results in abnormal flagella and reduced fertility in *mdx* ^*3cv*^; another mouse model of DMD whose AT to A transversion in exon 66 of *Dmd* causes frameshift of the encoded mRNA^18^. Although no altered fertility in mice lacking Dp427 (*mdx*) or utrophin (*utrn* ^−/−^) has been reported, *mdx/utrn* ^−/−^ mice null for both dystrophin and utrophin are infertile^19, 20^. In this study, we used *mdx* and *mdx/utrn* ^+/−^ mice to unravel the role played by dystrophin and utrophin in the male reproductive system.

## Results

### The *mdx/utrn*^+/−^ mice have impaired reproductive system

**Figure 1:**
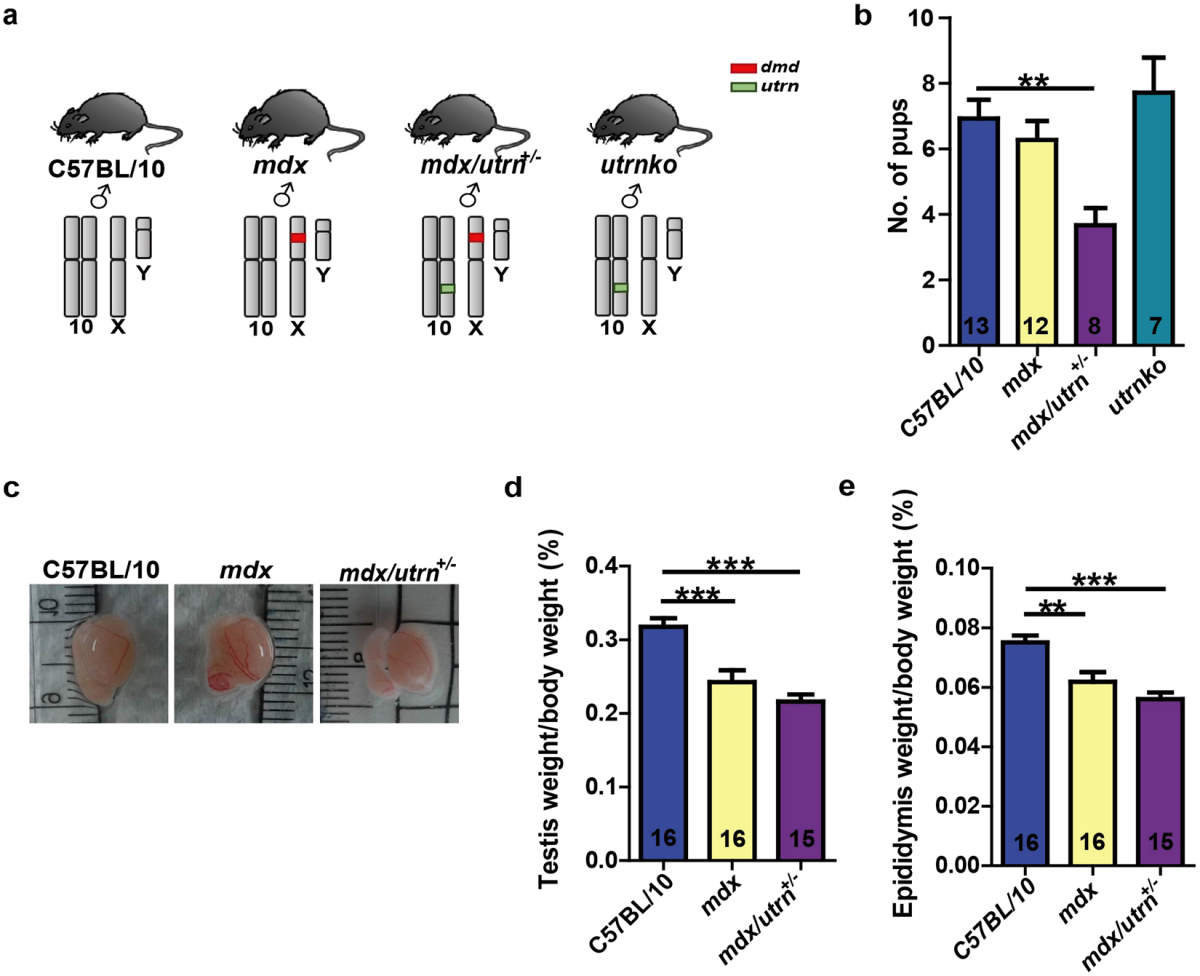
Dystrophin deficiency causes defects in the male reproductive organs. (**a**) The chromosome diagrams of mouse models for control (C57BL/10), dystrophin-deficiency (*mdx*), dystrophin-null/utrophin haplodeficiency (*mdx/utrn* ^+/−^) and utrophin null (*utrnko*). The red box indicates the mutant *dmd* locus whereas the green box indicates the mutated *utrn* locus. (**b**) The litter size in C57BL1/0, *mdx*, *mdx/utrn* ^+/−^ and *utrnko* mice. (**c**) Average testicle size in the ten-week-old C57BL/10, *mdx* and *mdx/utrn* ^+/−^ *mice*. (**d**) Statistical analysis of testis size of the ten-week-old C57BL/10, *mdx* and *mdx/utrn* ^+/−^ mice. (**e**) The statistical analysis of the epididymis size of the ten-week-old C57BL/10, *mdx* and *mdx/utrn* ^+/−^. Data were analyzed with one-way ANOVA. All values are represented as mean ± SEM. The number of mice examined was labeled on the bar charts. *compared with the C57BL/10. **P < 0.01; ***P < 0.001.

The *mdx/utrn* ^−/−^ (*dystrophin*- and *utrophin*-deficient) mice show premature death^19^. Therefore, we used *mdx* and *mdx/utrn* ^+/−^ (*dystrophin*-deficient and *utrophin*-haplodeficient) mice to elucidate the role of dystrophin and utrophin in male mouse reproductive system (Fig. 1a). The C57BL/10 and *mdx* mice gave birth to similarly-sized litters; however, the average number of pups per litter from *mdx/utrn* ^+/−^ mice was only half of C57BL/10 (Fig. 1b). Further examination of the male reproductive organs showed that the relative weights of testis and epididymis of *mdx* mice were reduced by 24% and 18%, respectively, compared to C57BL/10; nevertheless, utrophin haplodeficiency did not further reduce the size of testis or epididymis in *mdx/utrn* ^+/−^ mice (Fig. 1c–e). In addition, the litter size of the utrophin homozygous null mice (*utrnko*) was also comparable to C57BL/10 and *mdx* (Fig. 1a,b). Although the results suggest the involvement of dystrophin and utrophin during the development of male reproductive organs, the contribution of utrophin in *mdx/utrn* ^+/−^ mouse infertility needs further investigation.

### Dystrophin and utrophin are expressed in the testes

**Figure 2:**
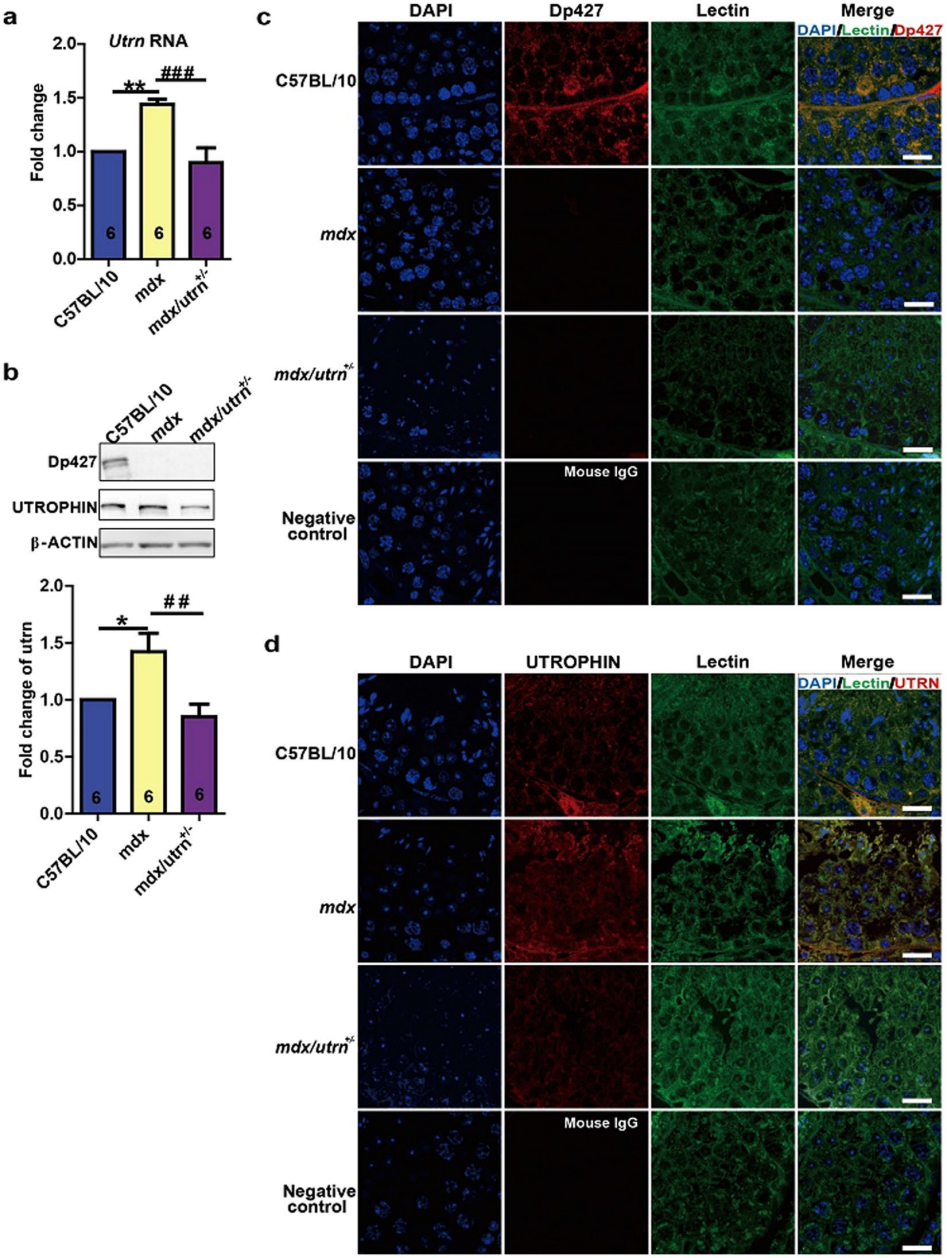
Dystrophin and utrophin express in the testes. Testes and epididymides of the ten-week-old mice were homogenized to isolate mRNA and protein for determining the expression of dystrophin and utrophin. (**a**) Quantitative PCR shows expression of utrophin in mice of all strains examined. (**b**) The upper panel shows the Western blotting for dystrophin and utrophin proteins in C57BL/10, *mdx* and *mdx/utrn* ^+/−^ testes. The lower panel shows the statistical analysis of the Western blotting for utrophin protein. The full blots of dystrophin and utrophin were provided in Supplementary Fig. S1a and b. (**c**,**d**) Cross-sections of the ten-week-old C57BL/10, *mdx* and *mdx/utrn* ^+/−^ testes were labeled with antibodies against dystrophin (**c**) and utrophin (**d**). The nuclei were tagged with DAPI and FITC-Lectin was used to outline the cells. utrn: utrophin. Scale bar: 20 μm. Data were analyzed with one-way ANOVA, *n* = 6. All values are represented as mean ± SEM. *compared with the C57BL/10; ^#^compared with the *mdx*. ^##^ *P* < 0.01; ^###^ *P* < 0.001; *P < 0.05; **P < 0.01.

Next, we analyzed the expression of dystrophin and utrophin in the testes. Quantitative real-time PCR (qPCR) revealed the expression of utrophin in the testes of C57BL/10, *mdx* and *mdx/utrn* ^+/−^ mice (Fig. 2a). Interestingly, the expression of utrophin rose 1.5 fold in *mdx* testes (Fig. 2a). Western blotting confirmed the absence of dystrophin protein in *mdx* and *mdx/utrn* ^+/−^ testes and a 1.5 fold increase of utrophin protein in the *mdx* testes (Fig. 2b and Supplementary Fig. S1a,b), suggesting a compensatory expression of utrophin in response to dystrophin deficiency. Immunofluorescent labeling showed the presence of full-length dystrophin (dystrophin with the molecular weight of 427 kDa and hereinafter referred to as Dp427) in the germ cells in C57BL/10 testes but not in *mdx* or *mdx/utrn* ^+/−^ testes (Fig. 2c). Similar to dystrophin in C57BL/10 testes, utrophin was also observed in the germ cells of C57BL/10, *mdx* and *mdx/utrn* ^+/−^ with more intense labeling for utrophin in *mdx* (Fig. 2d). These results indicate that dystrophin and utrophin may participate in the germ cell function.

### Spermatozoa from *mdx* and *mdx/utrn*^+/−^ have impaired mobility and morphology

**Figure 3:**
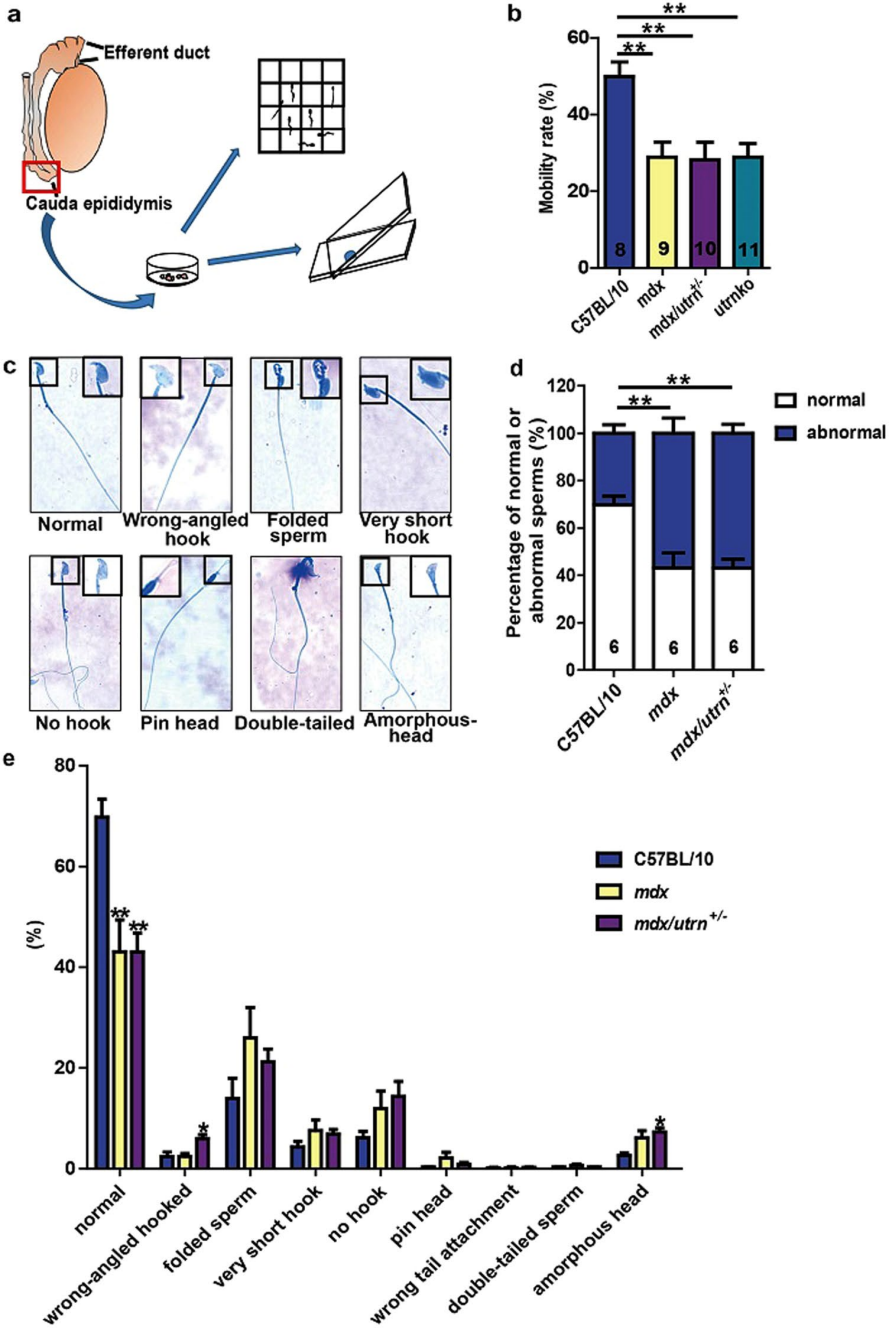
Dystrophin/utrophin deficiency impairs sperm mobility and morphology. (**a**) Schematic illustration showing procedure of isolating spermatozoa from the cauda epididymis of a ten-week-old mouse, counting spermatozoa numbers and spreading spermatozoa on the slides. (**b**) The percentage of motile spermatozoa in the cauda epididymidis of the C57BL1/0, *mdx*, *mdx/utrn* ^+/−^ and *utrnko* mice. (**c**) Representative spermatozoa morphology of the *mdx*, including normal, folded spermatozoa and spermatozoa with a wrong-angled hook, very short hook, no hook, pin head, amorphous head and double tails. (**d**) The percentage of spermatozoa with morphological abnormality in the cauda epididymidis of C57BL/10, *mdx* and *mdx/utrn* ^+/−^ mice. (**e**) The percentage of spermatozoa with different abnormal morphologies in the cauda epididymidis of the C57BL/10, *mdx* and *mdx/utrn* ^+/−^ mice. More than 500 spermatozoa were counted from each mouse to determine the sperm morphology. At least six mice of each strain were examined. Data were analyzed with one-way ANOVA. All values are represented as mean ± SEM. *compared with the C57BL/10; *P < 0.05; **P < 0.01.

We isolated spermatozoa from the cauda epididymidis of C57BL/10, *mdx* and *mdx/utrn* ^+/−^ mice in order to examine the influence of dystrophin/utrophin on spermatozoa (Fig. 3a). The motility rate of spermatozoa was determined as the percentage of spermatozoa with normally beating flagella. Nearly 50% of C57BL/10 spermatozoa were found to be motile; whereas the mobility rate of the spermatozoa from *mdx* or *mdx/utrn* ^+/−^ mice was only 30% (Fig. 3b and Supplementary Fig. S2a). Furthermore, the *utrnko* spermatozoa exhibited comparable motility rate as the *mdx* and *mdx/utrn* ^+/−^ spermatozoa (Fig. 3b). Aberrant spermatozoal morphologies such as folding or double tails influence their mobility, thus we next explored the spermatozoal morphology (Fig. 3c). By contrast to C57BL/10 spermatozoa, which show 30% of abnormality, both *mdx* and *mdx/utrn* ^+/−^ had up to 56% of the spermatozoon with abnormal morphology (Fig. 3d). Despite a comparable percentage of morphological abnormality in the spermatozoa of *mdx* and *mdx/utrn* ^+/−^ mice, the *mdx* spermatozoa had similar percentages of abnormality in the tail and the head (26.8 ± 5.9% vs. 30.1 ± 6.3%; p = 0.71) while the *mdx/utrn* ^+/−^ spermatozoa had a higher percentage of abnormality in the head over the tail (35.2 ± 3.8% vs. 21.7 ± 2.6%; p = 0.015) (Fig. 3d,e). Collectively, these data suggest both Dp427 and utrophin may be required for normal production of spermatozoa.

**Figure 4:**
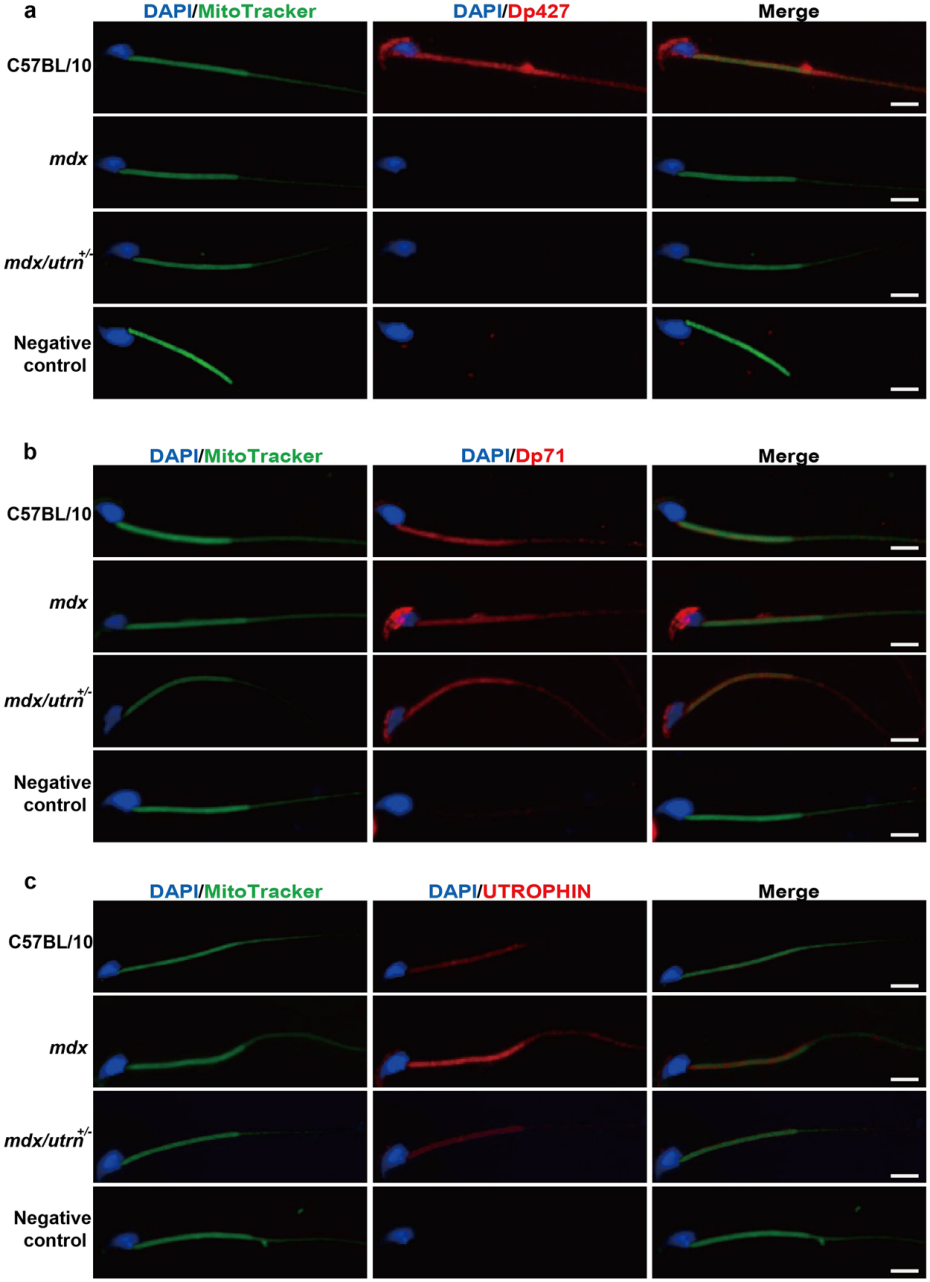
Upregulation of utrophin in response to dystrophin deficiency in spermatozoa. Spermatozoa were isolated from C57BL/10, *mdx* and *mdx/utrn* ^+/−^ at 10 weeks of age to determine the localization of Dp71, Dp427 and utrophin. The midpiece of spermatozoa were labeled with MitoTracker^®^ Green and the nuclei were labeled with DAPI. (**a**) Localization of Dp427 in the spermatozoa of C57BL/10 (the 1^st^ panel), *mdx* (the 2^nd^ panel) and *mdx/utrn* ^+/−^ (the 3^rd^ panel). (**b**) Localization of Dp71 in the spermatozoa of C57BL/10 (the 1^st^ panel), *mdx* (the 2^nd^ panel) and *mdx/utrn* ^+/−^ (the 3^rd^ panel). (**c**) Distribution of utrophin in the spermatozoa of C57BL/10 (the 1^st^ panel), *mdx* (the 2^nd^ panel) and *mdx/utrn* ^+/−^ (the 3^rd^ panel). The 4^th^ panels of (**a**–**c**) were Negative control. Scale bar: 5 μm.

Dp71 is the shortest and the predominant dystrophin isoform in the spermatozoa, whose expression affects the flagellum function and thus fertility^18^. To evaluate whether absence of the Dp427 affects Dp71 in spermatozoa, we immunofluorescently labeled Dp427, Dp71 and utrophin in spermatozoa isolated from 10-week-old C57BL/10, *mdx* and *mdx/utrn* ^+/−^. Dp427 localized in the spermatozoon head and the flagellar midpiece in C57BL/10 and, as expected, was absent from the spermatozoa from *mdx* and *mdx/utrn* ^+/−^ (Fig. 4a). Consistent with the previous report, Dp71 localized in the head of the spermatozoa and the midpiece of the flagella in C57BL/10 (Fig. 4b)^18^. Moreover, we observed no altered localization of Dp71in spermatozoa from *mdx* and *mdx/utrn* ^+/−^ compared to C57BL/10 (Fig. 4b), suggesting that Dp427 exerts no effect on the expression and localization of Dp71 in spermatozoa. Interestingly, utrophin was also located in the flagellar midpiece of the spermatozoa from C57BL/10, *mdx* and *mdx/utrn* ^+/−^ mice and was upregulated in the *mdx* spermatozoa (Fig. 4c), arguing partial functional redundancy of utrophin and dystrophin in the spermatozoal flagella. Collectively, these data indicate that Dp427 and utrophin may affect the spermatozoal mobility.

### The *mdx* and *mdx/utrn*^+/−^ testes have degenerated seminiferous tubules

The testes are occupied by the seminiferous tubules within which the germ cells lie and undergo spermatogenesis. Using hematoxylin-eosin staining, we investigated the effects of dystrophin/utrophin deficiency on testicular morphology. The majority of the C57BL/10 seminiferous tubules were composed of layers of spermatogonia, preleptotene spermatocytes, pachytene spermatocytes, round spermatids and elongated spermatids which are characteristic of spermatogenesis at stage VII-VIII (Supplementary Fig. S3a,b)^21, 22^. The elongated spermatids in the lumen of seminiferous tubules were fewer in the *mdx* testes than in the C57BL/10 testes (Supplementary Fig. S3a,b,d,e). Similarly, the epididymisal lumen also showed a reduction of spermatozoa in *mdx* mice (Supplementary Fig. S3c, compared with Fig. S3f). In addition, the seminiferous tubules of the *mdx* testes exhibited mild focal vacuolation, suggestive of moderate degeneration of seminiferous tubules (Supplementary Fig. S3e; arrow). Interestingly, utrophin haplodeficiency worsened the degeneration of testicular seminiferous tubules in *mdx/utrn* ^+/−^; the *mdx/utrn* ^+/−^ testes showed not only prominent vacuolation in most tubules (Supplementary Fig. S3g; arrows), but also multinucleated giant cells (Supplementary Fig S3g; arrowheads), which may be attributed to degeneration and widening of intercellular bridges between germ cells^23^. There was also a severe loss of elongated spermatids and spermatozoa in the tubule lumen of *mdx/utrn* ^+/−^ testes and epididymidis respectively (Supplementary Fig. S3h,i). These data indicate the deficiency of dystrophin and utrophin may lead to degeneration of seminiferous tubules.

### The *mdx* and *mdx/utrn*^+/−^ mice have perturbed gene expression for spermatogenesis

**Figure 5:**
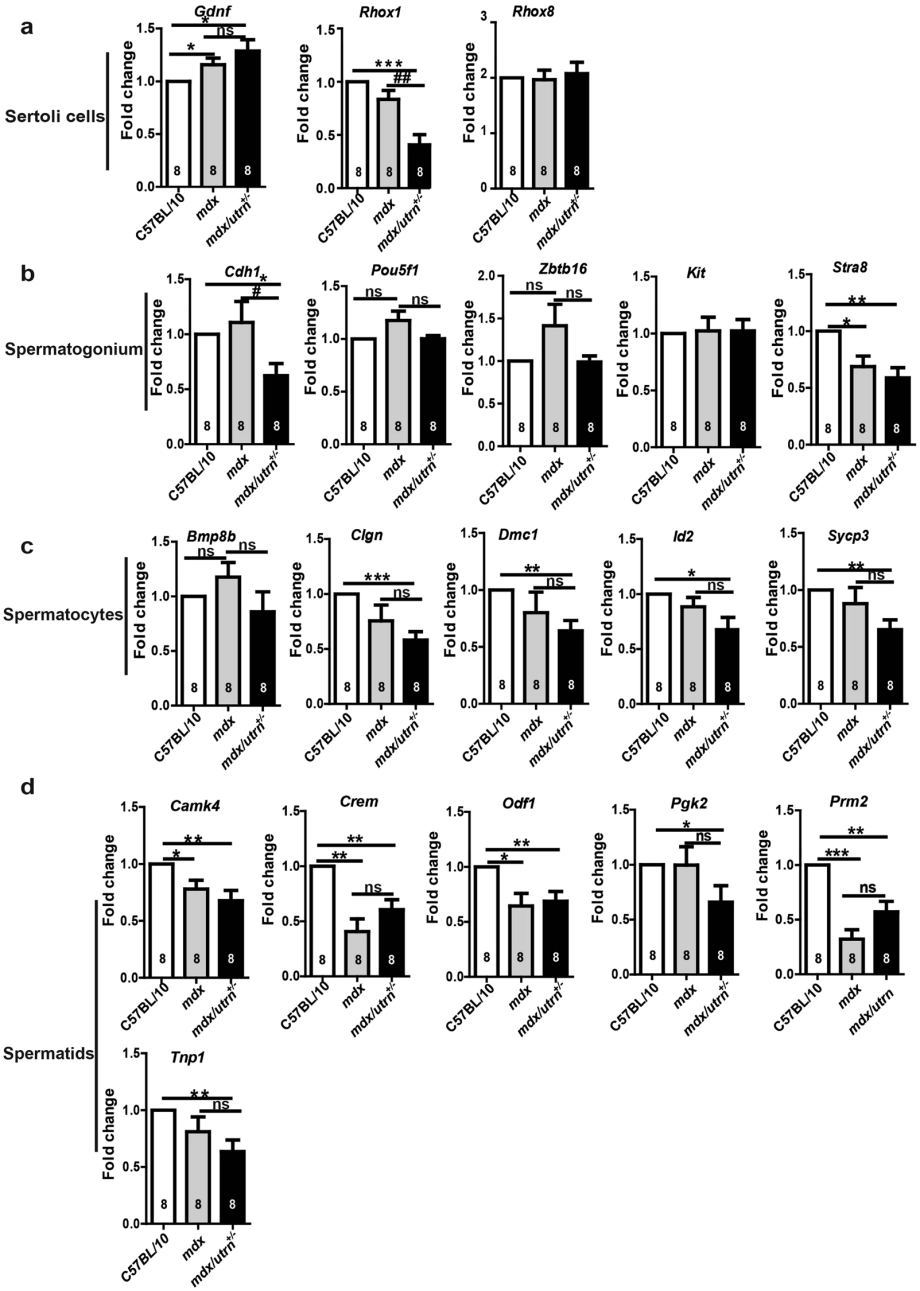
Deficiency of dystrophin/utrophin perturbs gene expression for spermatogenesis. The testes of the ten-week-old mice were homogenized for mRNA isolation. The expression of genes specific for germ cells of each stage was examined using quantitative PCR. (**a**) Sertoli cell-specific genes *Gdnf*, *Rhox1* and *Rhox8* in C57BL/10, *mdx* and *mdx/utrn* ^+/−^ testes. (**b**) The expression of genes specific for spermatogonia in C57BL/10, *mdx* and *mdx/utrn* ^+/−^ testes: *Cdh1*, *Kit*, *Pou5f1*, *Zbtb16* and *Stra8*. (**c**) Genes specific for spermatocytes in C57BL/10, *mdx* and *mdx/utrn* ^+/−^ testes: *Bmp8b*, *Clgn*, *Dmc1*, *Id2* and *Sycp3*. (**d**) Expression of the spermatid-specific genes in C57BL/10, *mdx* and *mdx/utrn* ^+/−^ testes: *CamK4*, *Crem*, *Odf1*, *Pgk2*, *Prm2* and *Tnp1*. Data were analyzed with one-way ANOVA; *n* = 6. All values are represented as mean ± SEM. ns: not significant. *compared with the C57BL/10; ^#^compared with the *mdx*. ^#^ *P* < 0.05; ^##^ *P* < 0.01; *P < 0.05; **P < 0.01; ***P < 0.001.

To establish whether the absence of dystrophin/utrophin influences Sertoli cells and germ cells of different stages, we evaluated the expression of genes specific to these cells^24^. Compared to C57BL/10, the expression of *Gdnf* was slightly increased in both *mdx* and *mdx/utrn* ^+/−^ Sertoli cells (Fig. 5a). Although the expression of Sertoli cell marker *Rhox1* in *mdx/utrn* ^+/−^ was only half that in C57Bl/10, the expression of another Sertoli cell marker *Rhox8* was constant in all three strains (Fig. 5a). The expression of the self-renewal related spermatogonium markers *Cdh1*, *Pou5f1* (also known as Oct4) and *Zbtb16* (also known as *Plzf*) was not significantly different in all three strains (Fig. 5b); however, *Stra8*, which is required for spermatogonium differentiation, was down-regulated in both *mdx* and *mdx/utrn* ^+/−^ (Fig. 5b). Except for *Bmp8b*, whose expression was not altered in spermatocytes of all three mouse strains, expression of the other spermatocyte markers *Clgn*, *Dmc1*, *Id2* and *Sycp3* decreased drastically in *mdx/utrn* ^+/−^ mice (Fig. 5c). With regard to the spermatids, there was a marked recession of *Camk4*, *Crem*, *Odf1*, *Pgk2*, *Prm2* and *Tnp1* in *mdx/utrn* ^+/−^ compared to C57BL/10 (Fig. 5d). Similarly, the expression of *Camk4*, *Crem*, *Odf1 and Prm2* was also decreased in *mdx* in comparison with C57BL/10 (Fig. 5d). The results argue that inadequacy of dystrophin/utrophin may contribute to perturbation of the progression through spermatogenesis in the testes.

### The number of spermatogenic cells diminishes in the *mdx* and *mdx/utrn*^+/−^ testes

**Figure 6:**
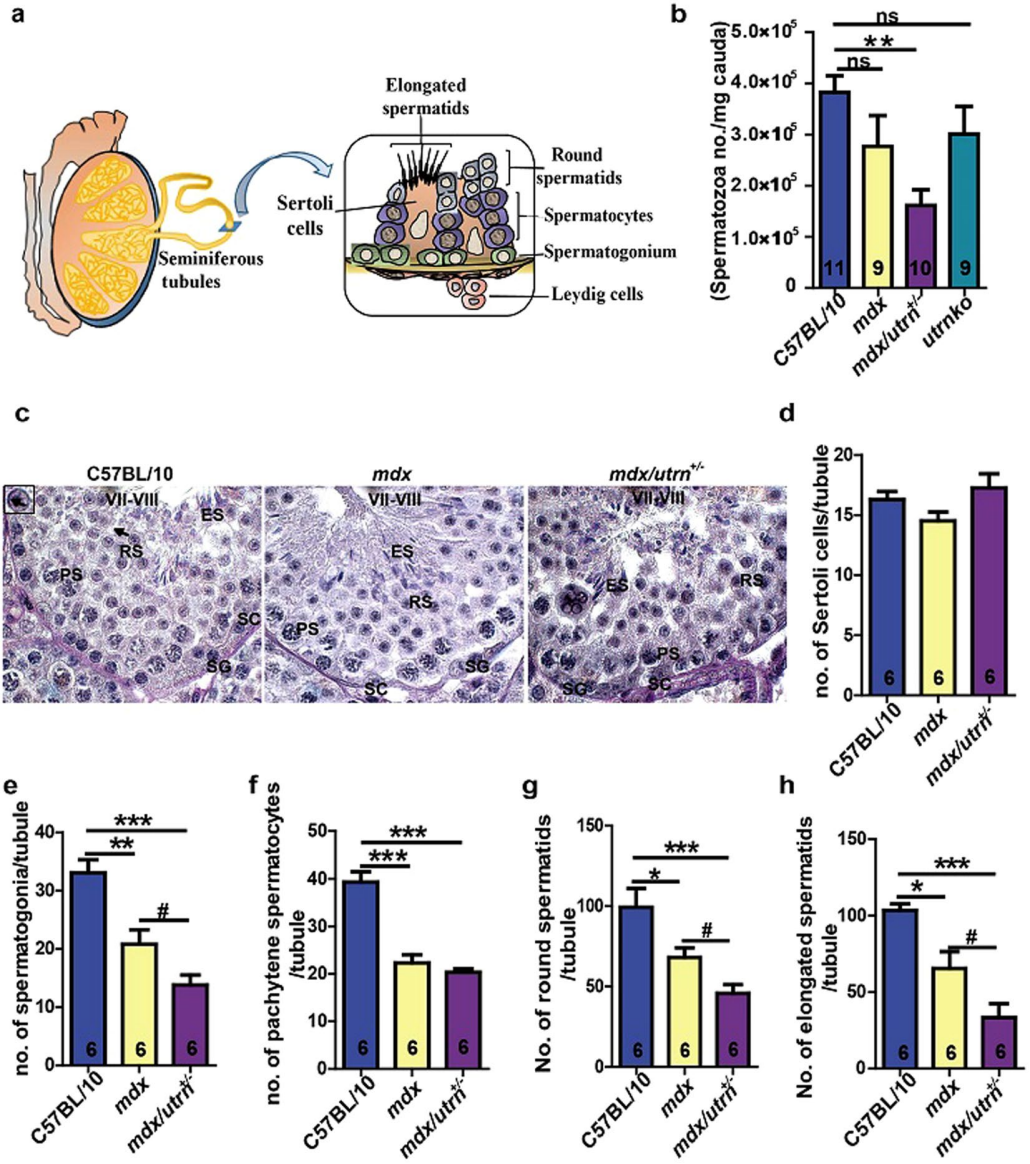
Deficiency of dystrophin/utrophin causes reduction of spermatogenic cells. (**a**) Schematic illustration of the structure of the seminiferous tubule which contains supporting Sertoli and Leydig cells, spermatogonia, spermatocytes, round spermatids and elongated spermatids. (**b**) The spermatozoa density of cauda epididymidis in C57BL/10, *mdx*, *mdx/utrn* ^+/−^ and *utrnko* mice. (**c**) The cross sections of the ten-week-old mouse testes were stained with PAS to classify germ cells of different stages. The arrows and inset in the first panel show the representative PAS staining of a round spermatid. (**d**) The number of Sertoli cells per seminiferous tubule in C57BL/10, *mdx* and *mdx/utrn* ^+/−^ testes. (**e**) The number of spermatogonia per seminiferous tubule in C57BL/10, *mdx* and *mdx/utrn* ^+/−^ testes. (**f**) The number of pachytene spermatocytes per seminiferous tubule in C57BL/10, *mdx* and *max/utrn* ^+/−^ testes. (**g**) The number of round spermatids per seminiferous tubule in C57BL/10, *mdx* and *mdx/utrn* ^+/−^ testes. (**h**) the number of elongated spermatids per seminiferous tubule in C57BL/10, *mdx* and *mdx/utrn* ^+/−^ testes. Data were analyzed with one-way ANOVA. All values are represented as mean ± SEM. *compared with the C57BL/10; ^#^compared with the *mdx*. ^#^ *P* < 0.05; *P < 0.05; **P < 0.01; ***P < 0.001. SC, Sertoli cells; SG, spermatogonium; PL, preleptotene spermatocyte; PS, pachytene spermatocyte; RS, round spermatid; ES, elongated spermatid. PAS: Periodic acid–Schiff stain; ns: not significant. Scale bar: 20 μm.

Spermatogenesis, a process involving differentiation of spermatogonia to spermatocytes and eventually the spermatids, initiates within the seminiferous tubules of the testis and requires the support of Sertoli and Leydig cells (Fig. 6a). To establish whether the deficiency of dystrophin/utrophin has an influence on germ cells, we focused on the changes of germ cell number. The spermatozoa concentration of the *mdx* and *utrnko* cauda epididymidis were less than that of C57BL/10 by 28% and 21% respectively though not statistically significant; moreover, the spermatozoa concentration further reduced by 58% in the *mdx/utrn* ^+/−^ cauda epididymis compared to C57BL/10 (Fig. 6b). In addition, there was a dramatic decrease of viable spermatozoal concentration determined with computer-assisted sperm assay in the *mdx* cauda epididymis (1.27 ± 0.12 million/ml) compared to C57BL/10 (8.17 ± 0.42 million/ml); the viable spermatozoal concentration further decreased in the *mdx/utrn* ^+/−^ epididymis (Supplementary Fig. 2b), reflecting that both dystrophin and utrophin play a role during spermatogenesis. We then investigated the average number of germ cells per seminiferous tubules at spermatogenic stages VII-VIII to determine the effects of dystrophin/utrophin insufficiency on spermatogenesis (Fig. 6c). The number of Sertoli cells per seminiferous tubule did not differ among C57BL/10, *mdx* and *mdx/utrn* ^+/−^ testes (Fig. 6c,d). However, the number of spermatogonia per seminiferous tubule decreased from 33.08 ± 2.24 in C57BL/10 to 20.78 ± 2.52 in *mdx* and further to 13.80 ± 1.696 in *mdx/utrn* ^+/−^ (Fig. 6c,e). In addition, the number of pachytene spermatocytes per seminiferous tubule in *mdx* (20.78 ± 2.52) and *mdx/utrn* ^+/−^ (13.80 ± 1.70) exhibited 37% and 58% reductions, respectively, compared to C57BL/10 (33.08 ± 2.24) (Fig. 6c,f). The number of round and elongated spermatids per seminiferous tubule reduced by 32% and 37% respectively in *mdx* by contrast to C57BL/10 (99.30 ± 11.72 for round spermatids and 103.50 ± 4.13 for elongated spermatids) (Fig. 6c,g,h). Moreover, deficiency of dystrophin/utrophin further declined the number of round and elongated spermatids per seminiferous tubules in *mdx/utrn* ^+/−^ by 54% and 68% respectively in comparison to C57BL/10 (Fig. 6c,g,h). These results demonstrate that dystrophin deficiency compromises spermatogenesis and concomitant utrophin haplodeficiency further exacerbates germ cell loss.

### The *mdx* and *mdx/utrn*^+/−^ mice have increased apoptosis and decreased proliferation of spermatogenic cells

**Figure 7:**
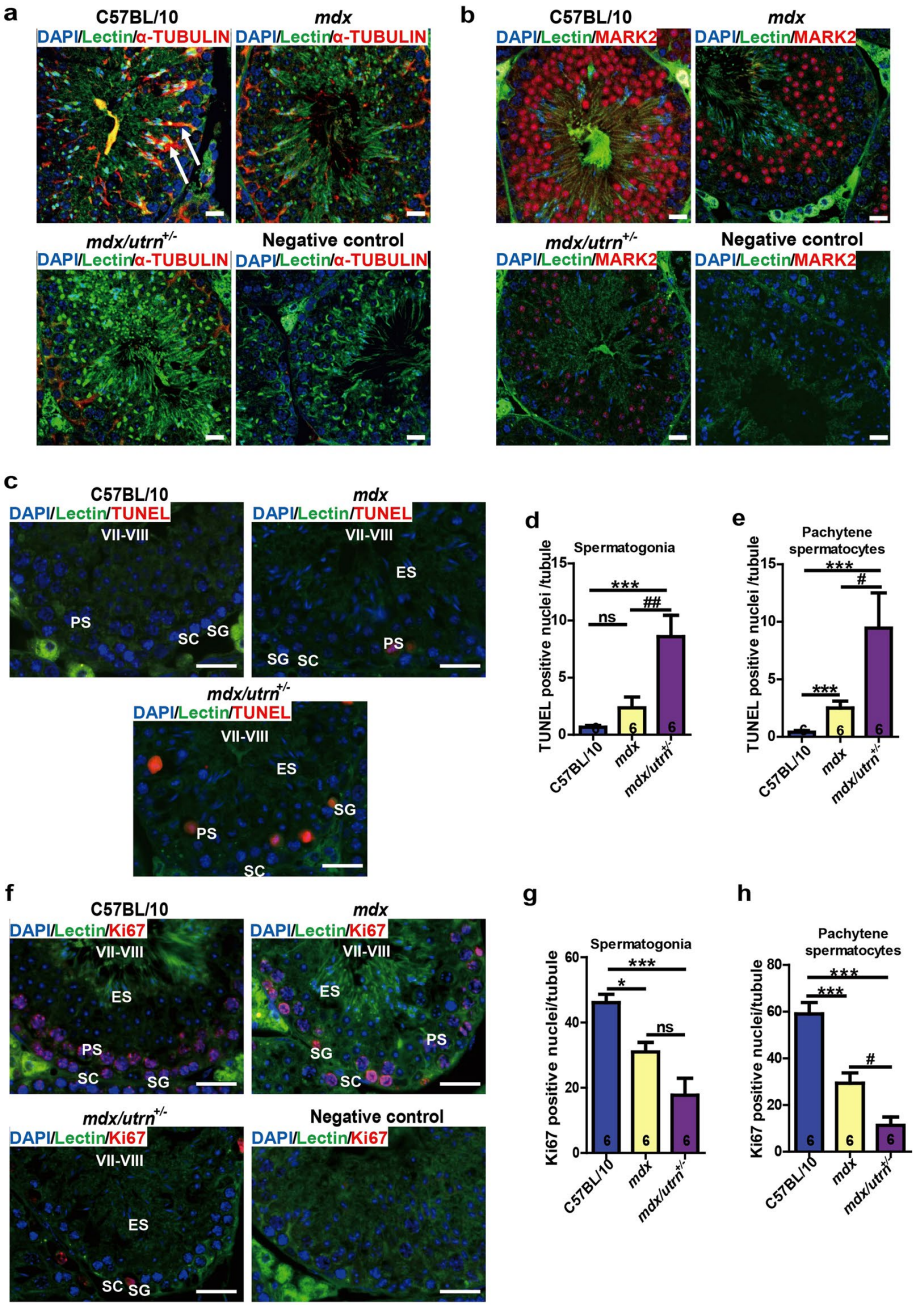
Utrophin haplodeficiency aggravates apoptosis raise and proliferation reduction of dystrophin-deficiency mouse spermatogenic cells. (**a**) The microtubule protrusion of the Sertoli cells in C57BL/10, *mdx* and *mdx/utrn* ^+/−^ testes. (**b**) MARK2 positive cells in C57BL/10, *mdx* and *mdx/utrn* ^+/−^ testes. (**c**) Cross-sections of ten-week-old mouse testes were labeled with TUNEL to identify the apoptotic cells. (**d**) The number of TUNEL-positive spermatogonia per seminiferous tubule in C57BL/10, *mdx* and *mdx/utrn* ^+/−^ testes. (**e**) The number of TUNEL-positive pachytene spermatocytes per seminiferous tubule in C57BL/10, *mdx* and *mdx/utrn* ^+/−^ testes. (**f**) Proliferating cells were labeled with Ki67 in the cross-sections of ten-week-old mouse testes. (**g**) The number of the Ki67-positive spermatogonia per seminiferous tubule in C57BL/10, *mdx* and *mdx/utrn* ^+/−^ testes. (**h**) The number of Ki67-positive pachytene spermatocytes per seminiferous tubule in C57BL/10, *mdx* and *mdx/utrn* ^+/−^ testes. MARK2: microtubule affinity regulating kinase 2; ns: not significant. Data were analyzed with one-way ANOVA, *n* = 6. All values are represented as mean ± SEM. *compared with the C57BL/10; ^#^compared with the *mdx*. ^#^ *P* < 0.05; ^##^P<0.01; *P < 0.05; ***P < 0.001. Scale bar: 50 μm.

The reduction of spermatogenic cells in *mdx* and *mdx/utrn* ^+/−^ could be attributed to increment in apoptosis and/or decline in proliferation. Loss of dystrophin in satellite cells results in downregulation of the serine-threonine kinase MARK2 (a cell polarity regulator), leading to defects in asymmetric division and prolonged cell cycle of satellite cells^7^. In the normal testes, Sertoli cells extend from the basal to the luminal compartment of the seminiferous tubules to support, nourish and organize spermatogenic cells^25, 26^. We first stained microtubules in the testes to identify the morphological changes of Sertoli cells. Protrusion of Sertoli cells was less in *mdx* and nearly loss in *mdx/utrn* ^+/−^ by contrast to that in C57BL/10 (Fig. 7a), which may lead to disorganization and loss of germ cells. We also observed significant decline of MARK2 positive germ cells in *mdx* and *mdx/utrn* ^+/−^, especially the *mdx/utrn* ^+/−^ (Fig. 7b), indicating impaired polarity of spermatogenic cells undergoing cell division.

We then evaluated whether deficiency on dystrophin/utrophin affects the rates of apoptosis and proliferation by TUNEL (Terminal deoxynucleotidyl transferase dUTP nick end labeling) assay and Ki67 (a nuclear protein associated with cell proliferation) staining respectively. The seminiferous tubules examined were at the stage VII-VIII of spermatogenesis. The number of TUNEL-positive spermatogonia and pachytene spermatocytes per seminiferous tubule were 3.6 fold and 6.25 fold more in *mdx* testes than in C57BL/10 respectively (Fig. 7c,d,e). Furthermore, 13.2 fold more TUNEL-positive spermatogonia and 23.6 fold more TUNEL-positive spermatocytes were observed in *mdx/utrn* ^+/−^ testes than in C57BL/10 respectively (Fig. 7c,d,e), suggesting that utrophin haplodeficiency further exacerbates germ cell apoptosis in the absence of dystrophin. On the other hand, the number of Ki67-spermatogonia and spermatocytes per seminiferous tubule in the *mdx* testes were only 67% and 50% that in C57BL/10 (46.08 ± 2.56 for spermatogonia and 58.98 ± 4.87) respectively (Fig. 7f,g,h). In addition, a further reduction in the number of Ki67-positive spermatogonia and spermatocytes per seminiferous tubule was observed in the *mdx/utrn* ^+/−^ testes which were only 38% and 19% that in C57BL/10 (Fig. 7f,g,h), indicating that dystrophin/utrophin deficiency also depleted proliferating germ cells. The results argue that both dystrophin and utrophin are required for spermatogenesis and that utrophin-haplodeficiency further exacerbates the effects of dystrophin deficiency on apoptosis and proliferation of germ cells.

## Discussion

In addition to the skeletal and cardiac muscles, dystrophin and utrophin are also expressed in the adult testes, in which their function remains unclear^9, 12^. Our goal was to investigate the role of dystrophin and utrophin in male reproductive system. In DMD patients, the level of utrophin negatively correlates with the disease severity^11, 17^. In the *mdx* mice, transgenic expression of both full-length and truncated utrophin lacking most spectrin repeats restores the components of dystrophin-glycoprotein complex to the sarcolemma to ameliorate the degeneration of myofibers and improves the muscle function^27–29^. All of these reports suggest a compensatory role of utrophin for dystrophin in skeletal muscles. In this report, we have demonstrated that dystrophin deficiency in the testes also up-regulates utrophin in a similar manner to the skeletal muscle (Fig. 2)^15, 17^, which appears to play a compensatory role. Interestingly, we found that dystrophin deficiency and utrophin haplodeficiency compromise fertility of male *mdx/utrn* ^+/−^ mice (Fig. 1). Consequently, we demonstrated perturbed spermatogenesis in male *mdx/utrn* ^+/−^ mice.

Previously, Hernandez-Gonzalez *et al*. showed the expression of Dp71 in the spermatozoal head and the midpiece of flagellum where it tightly regulates the level and localization of voltage-dependent Na^+^ (μ1), K^+^ (Kv1.1) channels and neural nitric oxide synthase (nNOS), and therefore the flagellar movement^18^. In this study, we demonstrated that Dp427 also participates in modulating the flagella function of spermatozoa (Figs 3 and 4). However, whether dystrophin performs its function in the flagella of spermatozoa via association with nNOS and voltage-dependent Na^+^ (μ1) and K^+^ (Kv1.1) channels has yet to be determined. One intriguing finding is that utrophin is not only located in the midpiece of the flagella but its expression is also upregulated in the absence of Dp427 (Fig. 4c), indicating that both utrophin and dystrophin play roles in the spermatozoal flagella. Although the motility rate of *utrnko* spermatozoa also decreased to a similar degree as *mdx* spermatozoa, neither the spermatozoal morphology nor the spermatozoal motility was further deteriorated in *mdx/utrn* ^+/−^ (Fig. 3 and Supplementary Fig. S2a) which suggest that dystrophin and utrophin may affect the spermatozoal motility through different mechanisms. One possible explanation why the spermatozoal motility was neither further exacerbated in *mad/utrn* ^+/−^ and nor restored in *mdx* may be due to the distinct binding properties of dystrophin and utrophin to the cytoskeleton. *In vitro*, binding of utrophin to actin filaments renders more restriction to the dynamics of actin filaments than the dystrophin does^30^. Furthermore, utrophin fails to restore the microtubule disorganization resulted from dystrophin deficiency in the *mdx* skeletal muscle^31^.

Another interesting discovery is that impaired spermatogenesis would be due to the dysfunction of Sertoli cells and the direct effect of dystrophin/utrophin deficiency on germ cells. *Rhox1* expression in Sertoli cells correlates with the proliferation of both Sertoli cells and spermatogonia^32^. We observed downregulation of *Rhox1* (Fig. 5a) and severe impairment of microtubule-mediated expansion of Sertoli cells in *mdx/utrn* ^+/−^ (Fig. 7a), suggesting that lack of dystrophin and utrophin leads to the failure of Sertoli cells to support proliferation and survival of germ cells. This is consistent with the previous reports showing that dysregulation of microtubules in the Sertoli cells accounts for the loss of germ cells^25, 26^. Moreover, downregulation of the undifferentiated spermatogonia marker *Cdh1* and differentiating spermatogonia marker *Stra8* in *mdx/utrn* ^+/−^ (Fig. 5b) correlates with the decrease in the number of spermatogonia in the *mdx/utrn* ^+/−^ seminiferous tubules (Fig. 6e)^33, 34^. Likewise, downregulation of the markers for spermatocytes (*Clgn*, *Dmc1*, *Id2* and *Sycp3*) and spermatids (*Camk4*, *Crem*, *Odf1*, *Pgk2*, *Prm2* and *Tnp1*) in *mdx/utrn* ^+/−^ (Fig. 5c,d) is also coincident with the decrease in the number of spermatocytes and spermatids in the *mdx/utrn* ^+/−^ seminiferous tubules (Fig. 6f–h). Although most of the germ cell markers did not alter in *mdx* (Fig. 5) the number of spermatogonia and spermatids decreased significantly in *mdx* compared to C57BL/10 (Fig. 6e,g,h), suggesting that dystrophin also involves in the progress of spermatogenesis. One thing noteworthy is that the number of germ cells declined further in *mdx/utrn* ^+/−^, indicating that up-regulation of utrophin in *mdx* may partially compensate the role of dystrophin during spermatogenesis.

The decrease in the germ cell number may be attributed to the decreased proliferation and/or increased apoptosis of the germ cells. In the present study, we demonstrated that deficiency of dystrophin and utrophin results in arrest of germ cell proliferation by showing a decrease in Ki67-positive and microtubule associated protein MARK2-positive spermatogenic cells (Fig. 7b,f–h), considering that MARK2 associates with dystrophin and utrophin and loss of dystrophin hinders the polarity of MARK2 and consequent catastrophe of asymmetric division of satellite cells^7^. In addition, we also showed the contribution of increased apoptosis in germ cells to the reduction of germ cells in *mdx* and *mdx/utrn* ^+/−^ (Fig. 7c–e). Collectively, our data demonstrated the requirement of dystrophin and utrophin during spermatogenesis through regulating the balance between proliferation and apoptosis.

In conclusion, we demonstrated in this report that (1) Dp427 affects the spermatozoal motility; (2) germ cell survival and proliferation require dystrophin and utrophin and (3) utrophin compensates for dystrophin deficiency during spermatogenesis but not on spermatozoal motility.

## Methods

### Animals

C57BL/10SnJ (C57BL/10), C57BL/10ScSn-*Dmd* ^*mdx*^/J (*mdx* mice), and Utrn^*tm1Jrs*^Dmd^*mdx*^/J (*mdx/utrn* ^+/−^ mice) mice were purchased from the Jackson Laboratory (Bar Harbor, ME, USA). The C57BL/10 mice were first crossbred with *mdx/utrn* ^+/−^ mice to obtain the *utrn* ^+/−^ (utrophin heterozygous knockout) mice, and the F1 *utrn* ^+/−^ mice were interbred to obtain the homozygous utrophin null mice, the *utrnko* mice. All experiments were in accordance with the Guides for the Use and Care of Laboratory Animals (ARRIVE guidelines), and the animal protocols have been approved by Experimental Animal Committee, Academia Sinica, Taiwan. The mice were housed in individually ventilated cages (IVCs) system in Academia Sinica SPF Animal Facility. The average litter size was determined by mating three pairs of each strain.

### Mouse testes and epididymides size determination

The body weights of the male mice were measured on a Toledo Precision Balance (Mettler Toledo, Switzerland). Sedentary male mice were sacrificed and the testes and epididymides were dissected at the age of 10 weeks. The weights of the testes and epididymides were measured on a Toledo Analytical Balance (Mettler Toledo, Switzerland). The sizes of the testes and epididymides were presented as the ratio to the body weight (%).

### Mouse sperm preparation

To evaluate the cauda epididymal spermatozoal concentration and motility, fresh cauda epididymidis were dissected from 10-week old male mice, placed into 2 ml of Whitten’s HEPES medium (100 mM NaCl, 4.4 mM KCl, 1.2 mM KH_2_PO_4_, 1.2 mM MgSO_4_, 5.4 mM glucose, 0.8 mM pyruvate, 4.8 mM lactic acid and 20 mM HEPES) and kept in 5% CO_2_ at 37 °C. Each epididymis was minced to allow the spermatozoa to disperse in the medium. After 30 minutes, the spermatozoal suspension was collected and diluted for quantitative assessment. The number of motile and total sperms were determined using a Hemocytometer (Hausser Scientific, USA).

### Spermatozoal morphology analysis

The spermatozoa from 10-week-old C57BL/10, *mdx* and *mdx/utrn* ^+/−^ were fixed with 4% paraformaldehyde (Sigma-Aldrich, 158127) for 15 minutes, washed with 100 mM ammonium acetate (Merck, Darmstadt, DE) and spread on slides (Marienfeld-Superior, Lauda-Königshofen, DE). The spermatozoal spread was stained with 0.22% Coomassie blue (Sigma-Aldrich, B0770) in 50% Methanol (9070; Avantor Performance Materials, Center Valley, PA, USA) and 10% Acetic Acid (9375; Avantor Performance Materials, Center Valley, PA, USA) for 30 minutes and the morphology were examined under microscope. 6 mice of reach strain were examined and more than 500 spermatozoa from each mouse were scored. The spermatozoal morphology was classified according to the criteria used by Wyrobek *et al*.^35^ and Smith *et al*.^36^ as normal, folded, double-tailed, pin head, amorphous head or head with a wrong-angled hook, very short hook or no hook^35, 36^.

### Histology and immunofluorescent labeling

Tissues were fixed overnight in Bouin’s solution (Sigma-Aldrich, HT10132), paraffin-embedded and sectioned at 3 μm. Sections were subjected to hematoxylin-eosin stain and periodic acid–Schiff stain (Merck Millipore, Billerica, MA, USA) following manufacturer’s instructions. For immunofluorescent labeling, sections were first antigen retrieved in boiling 10 mM citrate buffer (pH = 6.0) for 12 minutes. The sections and the spermatozoal spreads were blocked with 10% goat serum (Gibco, Grand Island, NY, USA) and 10% fetal bovine serum (FBS; Gibco, Grand Island, NY, USA). The following primary antibodies were used for detecting full length dystrophin Dp427 (MANDYS1 3B7; 1:500; Wolfson CIND, OSWESTRY, UK), Dp71 (MANDRA1 7A10; 1:100; Wolfson CIND, OSWESTRY, UK), utrophin (MANCHO3 8A4; 1:100; Wolfson CIND, OSWESTRY, UK), α-tubulin (1:1000; Sigma-Aldrich, T9026), ki67 (1:500; GTX16667; GeneTex, Irvine, CA, USA) and MARK2 (1:100; GTX111783; GeneTex, Irvine, CA, USA). The spermatozoal spreads were also immunofluorescently labeled with MitoTracker® Green (200 nM; Invitrogen, Carlsbad, CA, USA). The following second antibodies were used to provide fluorescent signals: anti-mouse IgG-Alexa Flour 488 (1:1,000; Invitrogen, Carlsbad, CA, USA), anti-rabbit Igs-Alexa Flour 568 (1:1,000; Invitrogen, Carlsbad, CA, USA).

### TUNEL assay

The testes of the ten-week-old mice were fixed in Bouin’s Solution, paraffin-embedded and sectioned at 3 μm. Levels of germ cell apoptosis were measured by terminal deoxynucleotidyl transferase dUTP nick end labeling (TUNEL) assay using ApopTag® Plus *In Situ* Apoptosis Fluorescein Detection Kit (Merck Millipore, Billerica, MA, USA). DNA breaks were labeled with fluorescein and visualized with Rhodamine.

### Separation of the testicular germ cell population

Separation of spermatogenic cells was based on the centrifugal elutriation system^37^. The testes of 10-week-old C57BL/10, *mdx* and *mdx/utrn* ^+/−^ mice were excised, decapsulated and cut into small pieces in dishes. Testes were incubated in 50 ml DMEM/F12 (Invitrogen, Carlsbad, CA, USA) with 1 mg/ml trypsin (Invitrogen, Carlsbad, CA, USA), 0.75 mg/ml collagenase (Invitrogen, Carlsbad, CA, USA), protein inhibitor (Sigma-Aldrich), DNase I (Sigma-Aldrich; 10104159001) and 1% penicillin/streptomycin (Sigma-Aldrich; P4333) and shacked at 180 rpm/minute for 90 minutes. The medium was loaded on 40 um filters (BD Bioscience, Sparks, MD, USA) and washed with DMEM/F12 medium. The cell suspensions were subjected to centrifugal elutriation to collect suspensions containing germs cells of different stages in the following sequence: 700 × g for 1 minute for spermatogonia, 400 × g for 1 minutes for round spermatids, 200 × g for 1 mins for secondary spermatocytes, 100 × g for 1 minutes for primary spermatocytes. The collected suspensions were centrifuged at max 3,000 × g for 10 minutes and the pellets were subjected to RNA purification using TRIZOAL reagent (Invitrogen, Carlsbad, CA, USA).

### Quantitative Real-Time PCR (qPCR)

Testes and epididymides were homogenized by MagNA lyser system (Roche, Basel, CH) and total RNA was extracted by TRIZOL reagent following manufacturer’s RNA extraction protocol. RNA was reversely transcribed by SuperScript III First-Strand Synthesis system (Invitrogen, Carlsbad, CA, USA) to generate cDNA in accordance with manufacturer’s instruction. In each qPCR reaction, the cDNA equivalent to 25 ng of RNA was used. Amplification of cDNA was monitored on an ABI 7500 real-time PCR system (Applied Biosystems, Foster City, CA, USA) with OmicsGreen qPCR Master Mix (Omics Bio, Taipei, Taiwan) and the forward and reverse primer each at a final concentration of 0.25 µM. The amplification protocol consisted of 2 minutes 50 °C and 10 minutes 95 °C followed by 50 cycles of 15 minutes 95 °C and 1 minute 60 °C; and completed with a standard melting curve protocol. Results were normalized to the expression of *Csnk2a2*. The mRNA primer sequences are detailed in Supplementary Table S1.

### Western blotting

The testes and epididymides of C57BL/10, *mdx* and *mdx/utrn* ^+/−^ were homogenized in RIPA buffer (50 mM HEPES, pH7.5; 140 mM NaCl; 1 mM EDTA; 1% Triton X-100 and 0.1% SDS) supplemented with protease inhibitor cocktail (Sigma-Aldrich) by MagNA lyser system. The lysate was then centrifuged at 15,800 × g for 10 minutes at 4 °C. 50 μg of protein lysate was denatured and separated using SDS-polyacrylamide electrophoresis following Dr. Thomas Krag’s protocol for separating dystrophin and utrophin^38^. Proteins were loaded on 5% polyacrylamide resolving gel directly and separated at 40 mA/gel in running buffer (192 mM glycine, 25 mM Tris base and 0.1% SDS) for 3–4 hours. Proteins were transferred to the PVDF membrane (PerkinElmer, Richmond, CA, USA) in transfer buffer (192 mM glycine, 25 mM Tris base, 15% methanol and 0.1% SDS) at 300 mA overnight. Dystrophin and utrophin were detected with anti-dystrophin (MANDYS1 3B7; 1:500; Wolfson CIND, OSWESTRY, UK) and anti-utrophin (MANCHO3 8A4; 1:100; Wolfson CIND, OSWESTRY, UK) respectively.

### Statistical analysis

Statistical analysis with GraphPad Prism 5 software (GraphPad Software, San Diego, CA, USA) was performed using the two-tailed unpaired Student’s t-test or one-way analysis of variance (ANOVA) followed by post hoc Tukey HSD (Honestly Significant Difference) multiple comparison tests. The results were recognized as statistically significant at *P* < 0.05. Asterisks and hashtags indicate the level of statistical significance compared with C57BL/10 and *mdx* respectively: *P < 0.05; **P < 0.01; ***P < 0.001; ^#^ *P* < 0.05; ^##^ *P* < 0.01; ^###^ *P* < 0.001; ns indicates not significant. The data were presented as means mean ± SEM.

## Declarations

### Ethics approval and consent to participate

All experiments were in accordance with the Institute of Animal Care and Use Committee of Academia Sinica, Taiwan. The IACUC reference number is 1109211.

## Electronic supplementary material

Supplementary information
