# Supplementary material for: Utrophin Compensates dystrophin Loss during Mouse Spermatogenesis
Source: Sci Rep. 2017 Aug 7;7:7372. doi: 10.1038/s41598-017-05993-8 (PMC5547154; doi:10.1038/s41598-017-05993-8)
Supplement: Supplementary file 1 — Supplementary information [file 41598_2017_5993_MOESM1_ESM.pdf]

Supplementary Information

Utrophin compensates dystrophin loss during mouse spermatogenesis

Hung-Chih Chen[1], Yu-Feng Chin[1], David J. Lundy[1], Chung-Tiang Liang[2], Ya-Hui Chi[3], Paolin Kuo[4], Patrick C.H.Hsieh[1,5]

[1]Institute of Biomedical Sciences, Academia Sinica, Taipei 115, Taiwan

[2]National Laboratory Animal Center, National Applied Research Laboratories, Taipei 115, Taiwan

[3]Institute of Biotechnology and Pharmaceutical Research, National Health Research Institutes, Zhunan, Miaoli County 350, Taiwan

[4]Department of Obstetrics & Gynecology, College of Medicine, National Cheng Kung University, Tainan 701, Taiwan

[5]Institute of Medical Genomics and Proteomics, Institute of Clinical Medicine and Cardiovascular Surgery Division, National Taiwan University and Hospital, Taipei 100, Taiwan

1 supplementary method
3 supplemental figures
1 supplemental table

Supplementary method:

Computer-assisted sperm analysis (CASA)

To evaluate the epididymal spermatozoal concentration and motility, fresh cauda epididymidis were dissected from 6-week old male mice, minced in 2 ml of Whitten's HEPES medium (100 mM NaCl, 4.4 mM KCl, 1.2 mM KH_2PO_4, 1.2 mM $MgSO_4$, 5.4 mM glucose, 0.8 mM pyruvate, 4.8 mM lactic acid and 20 mM HEPES) and kept in 5 % CO_2 at 37 °C to allow the spermatozoa to disperse in the medium for 30 minutes. The spermatozoal suspension was applied to a 10 µm (depth) Makler counting chamber (Sefi Medical Instrument; Haifa, Isreal) and the spermatozoal concentration (10^6/ml) and motility (%) were measured using computer-assisted sperm analysis (CASA) device with integrated visual optical system (IVOS) software (Hamilton-Thorne Research, Beverly, MA, USA). The percentages of motile spermatozoa, defined as spermatozoa moving at a path velocity (VAP) of 10 µm/s in any directions, and progressive motile spermatozoa, classified as spermatozoa having a VAP of more than 50 µm/s and a straightness ratio of more than 80%, were calculated. Videos of motile spermatozoa were captured with an Olympus IX-70 microscope and a 10x objective equipped with cooled charge-coupled device (CCD) digital camera (Olympus Optical Co. Ltd, Tokyo, Japan) at the frequency of 60 Hz. For each sample, eight fields were acquired and repeated three times. At least three different samples of each mouse strained were analyzed.

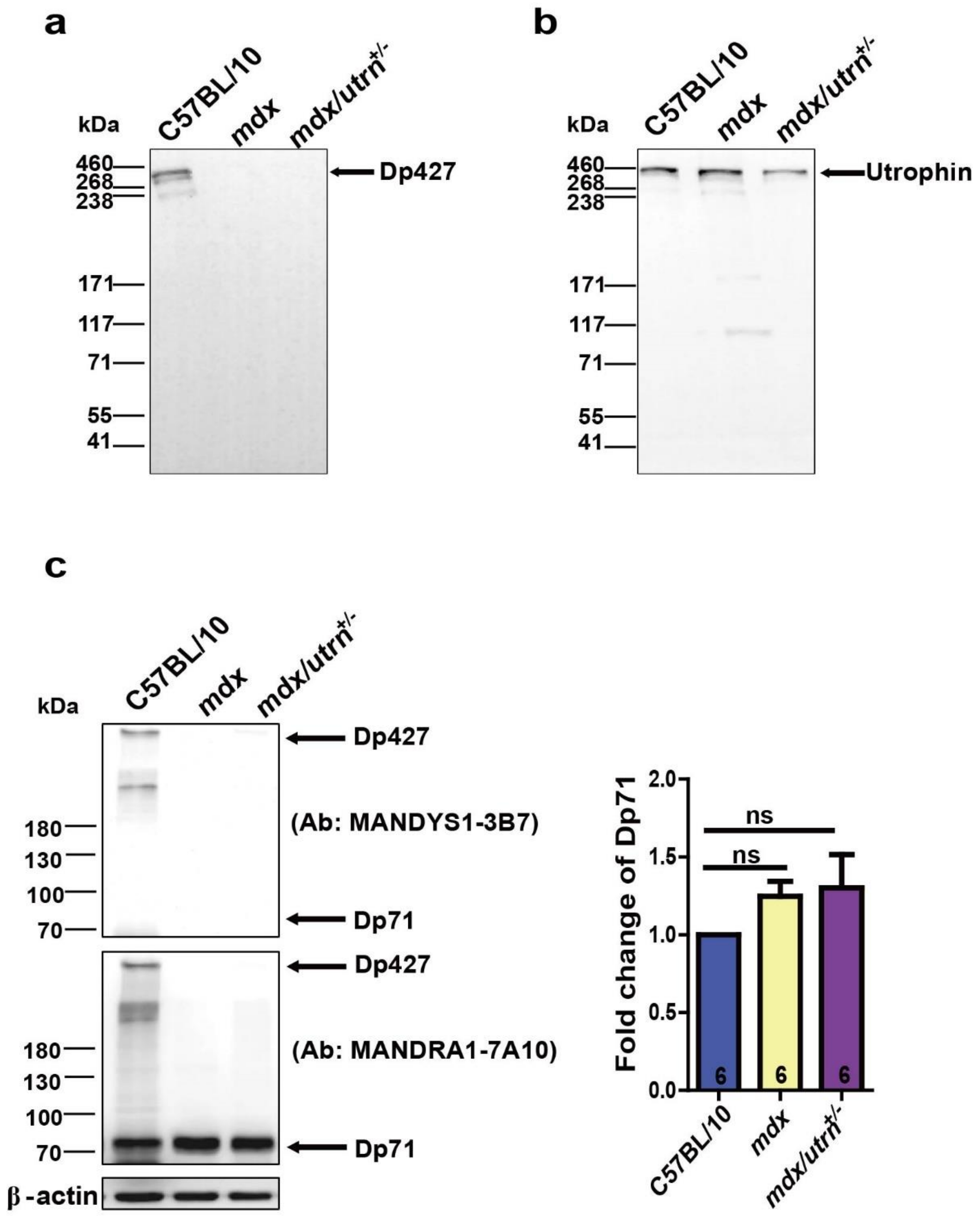

Figure S1 Expression of Dp427, utrophin and Dp71 in the 10-week-old C57BL/10, *mdx*, *mdx/utrn*$^{+/-}$ testes. (a)The full Western blotting image of Dp427 probed with MANDYS1-3B7 antibody and labeled with molecular weight markers for Fig 2b. (b) The full Western blotting image of utrophin probed with MANCHO3-8A4 antibody and labeled with molecular weight markers for Fig 2b. (c) Western blotting of Dp71 probed with MANDYS1-3B7 and MANDRA1-7A10 antibodies. Quantification of Dp71 blotting densitometry was normalized with β-actin. ns: not significant. Data were analyzed with one-way ANOVA, n=6.

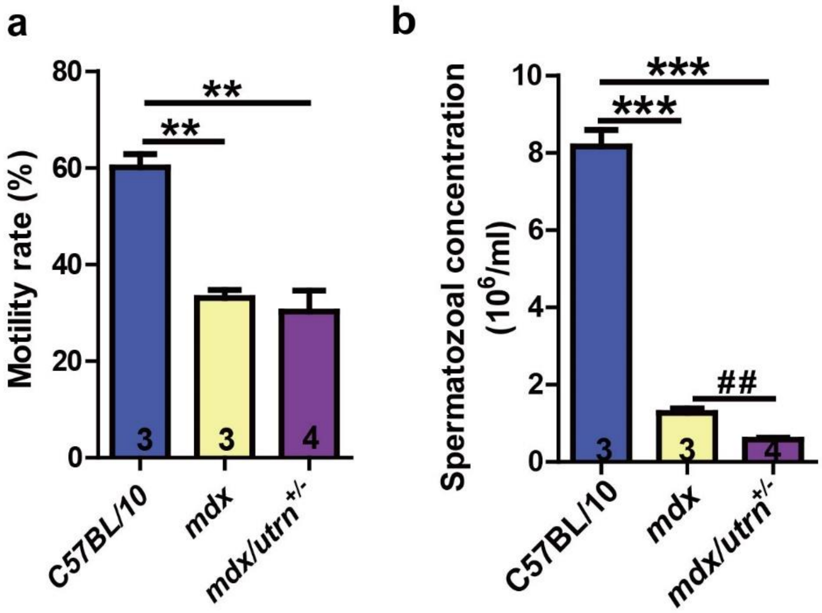

Figure S2 The spermatozoal concentration and motility of 6-week-old C57BL/10, *mdx*, *mdx/utrn*$^{+/-}$ mice. (a) The motility rate of 6-week-old C57BL/10, *mdx*, *mdx/utrn*$^{+/-}$ spermatozoa determined by computer-assisted sperm analysis (CASA). (b) The concentration of viable spermatozoa isolated from cauda epididymis of 6-week-old C57BL/10, *mdx*, *mdx/utrn*$^{+/-}$ mice using CASA. Data were analyzed with one-way ANOVA. The number of mice examined was labeled on the bar charts. All values are represented as mean±SEM. *compared with the C57BL/10; #compared with the mdx. ##P<0.01; **P<0.01; ***P<0.001.

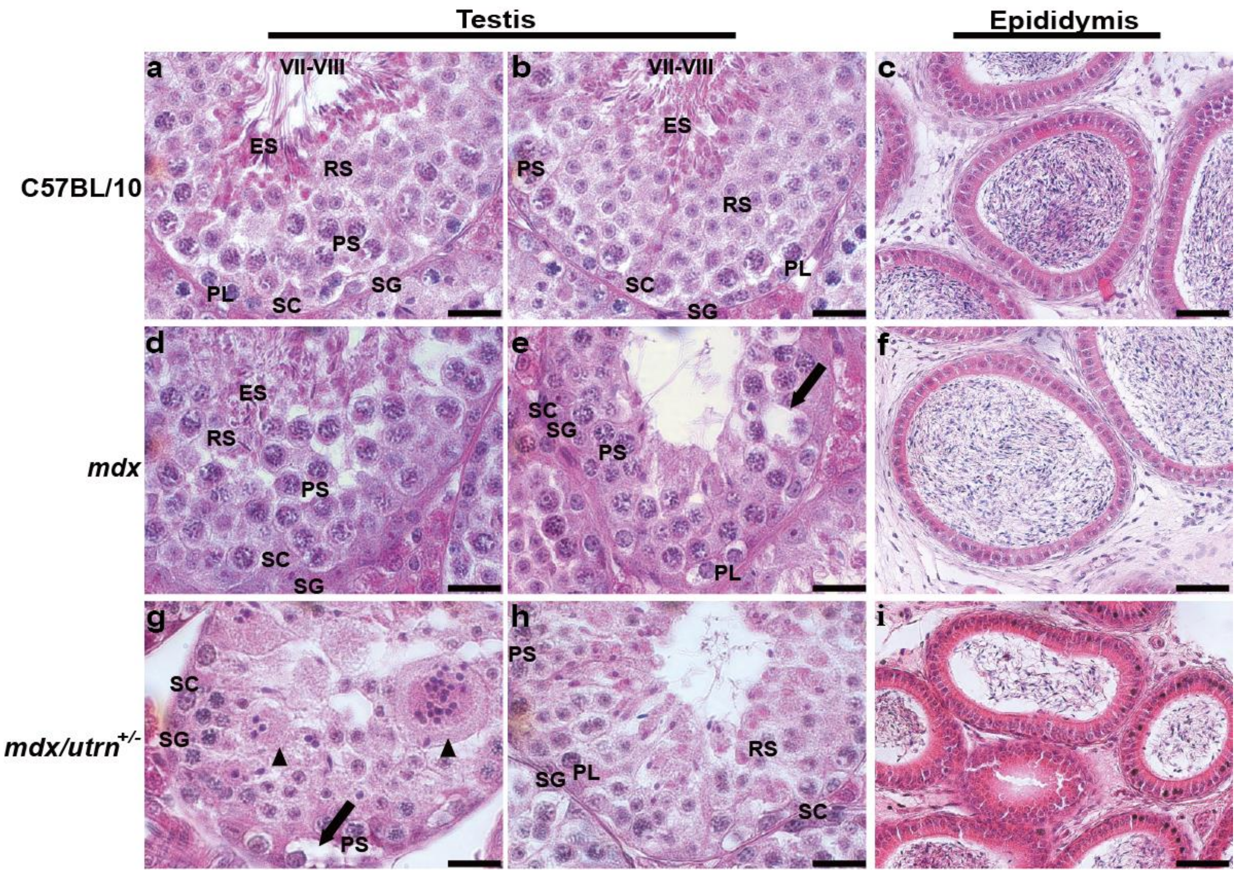

Fig. S3. Haplodeficiency of utrophin exacerbates degeneration of seminiferous tubules in the absence of dystrophin. Cross-sections of the ten-week-old mouse testes and epididymides were stained with hematoxylin-eosin for morphological analysis. **a**, **b** The seminiferous tubules of the C57BL/10 testis were mostly in stage VII-VIII. **c** The lumens of the C57BL/10 epididymis. **d**, **e** The seminiferous tubules of the *mdx* testis. **f** The epididymis lumens of the *mdx*. **g**, **h** The seminiferous tubules of the *mdx/utrn*$^{+/-}$. **i** The epididymis lumens of the *mdx/utrn*$^{+/-}$. Scale bar: 20 μm in (**a**, **c**, **d**, **f**, **g**, **i**); 40 μm in (**b**, **e**, **h**). Arrows indicate vacuolation of the seminiferous tubule and arrowheads indicate the multinucleated spermatocytes and spermatids. SC, Sertoli cells; SG, spermatogonium; PL, preleptotene spermatocyte; PS, pachytene spermatocyte; RS, round spermatid; ES, elongated spermatid.

Table S1. Primer sets for qPCR

Gene	Sense (5'-3')	Antisense (5'-3')
Bmp8b	TTTGACCTAACCCAGATCCC	AGATCGGAGCGTCTGAAGAT
Camk4	CCCATGGGTCACAGGTAAAG	TGTGGTTCTCTTGGATGCTG
Cdh1	ACGCTGAGCATGTGAAGAAC	CTGGATCCAAGATGGTGATG
Clgn	CTCATGTACCTGGTGATGGC	TGCACATCTTCTGGCTTCTC
Crem	GCGACAACCGCATCAGAG	TCCTTCCCTGTTTTCCTTATTT
Csnk2a2	TGTCCACCCTTACCTCTTCC	CCTCCCTCTCATTGGTTAGC
Dmc1	CAGCTCAACTTCCAGGAACA	GAAACTTGGCTGCGACATAA
GDNF	AATCGGCCGAGACAATGTA	TACATCCACACCGTTTAGCG
Id2	CCCGATGAGTCTGCTCTACA	TTCTGTCCAGGTCTCTGGTG
Kit	CAACTCTGATGCCAGTGCTT	ACCTGTACGTCCACTGGTGA
Oct4	GGCTGCTGCTTCTGTCTGT	CTGTCGGTTTCTCAGGCTTT
Odf1	CGTGAAAGATGGGAAGGTCT	GTGCAAGGGTAACATGGAGA
Pgk2	GCACTGTCTGGACAATGGAG	CATTATCTGGGTTGGCACAG
PLZF	GAGCACACTCAAGAGCCACA	GTGGCAGAGTTTGCACTCAA
PRM2	GGACTATGGGAGGACACACA	TCCTACATTTCCTGCACCTG
Rhox1	TTCGAGAGACATTCCTGTGG	GTTTCTTCCTTCTCCAGCCA
Rhox8	GGTTAAGCCCAGAATTTCCA	CAACAAAGGGCAGTCCTGTA
Scp3	ATCAGCAGAGAGCTTGGTCG	GTGGCTTCCCAGATTTCCCA
Stra8	GTCGATCTCTCCCACTCCTC	TCCTCAGAGAGAGTCTGCCA
Tnp1	AGAACCGAGCTCCTCACAAG	CTGATGTCCTCATGCTCCTG
Utrn	TCATGCTAGCCTGGACCATTTT	CACTGATGGGTGGTTTCCCA
